# *Trypanosoma cruzi* Induces Regulatory B Cell Alterations in Patients With Chronic Chagas Disease

**DOI:** 10.3389/fcimb.2021.723549

**Published:** 2021-08-12

**Authors:** Magalí C. Girard, Micaela S. Ossowski, Arturo Muñoz-Calderón, Marisa Fernández, Yolanda Hernández-Vásquez, Raúl Chadi, Karina A. Gómez

**Affiliations:** ^1^Laboratorio de Inmunología de las Infecciones por Tripanosomátidos (LIIT), Instituto de Investigaciones en Ingeniería Genética y Biología Molecular “Dr. Héctor N. Torres” (INGEBI), Consejo de Investigaciones Científicas y Técnicas (CONICET), Buenos Aires, Argentina; ^2^Laboratorio de Biología Molecular de la Enfermedad de Chagas (LabMECh), Instituto de Investigaciones en Ingeniería Genética y Biología Molecular “Dr. Héctor N. Torres” (INGEBI), Consejo de Investigaciones Científicas y Técnicas (CONICET), Buenos Aires, Argentina; ^3^Instituto Nacional de Parasitología “Dr. Mario Fatala Chabén”, Buenos Aires, Argentina; ^4^Hospital General de Agudos “Dr. Ignacio Pirovano”, Buenos Aires, Argentina

**Keywords:** regulatory B cells, IL-10-producing B cells, chronic Chagas disease, human *Trypanosoma cruzi* infection, chronic Chagas cardiomyopathy, PD-L1

## Abstract

The clinical evolution of patients with chronic Chagas disease (CCD) is mainly associated with an excessive inflammation and a defective immunomodulatory profile caused by the interaction between *T. cruzi* and the host. Regulatory B (Breg) cells exert immune suppression mostly through IL-10 production (B10 cells), but also through IL-10-independent mechanisms. Previously, we demonstrated that CCD patients with cardiomyopathy show changes in the *ex vivo* Breg cell phenotypic distribution although maintain IL-10 production capacity. Here, we sought to identify potential alterations on Breg cells upon *in vitro* stimulation. Isolated B cells from CCD patients with or without cardiomyopathy and non-infected (NI) donors were stimulated with *T. cruzi* lysate or CpG + CD40L, and characterized by flow cytometry based on the expression of CD24, CD27, CD38, and the regulatory molecules IL-10 and PD-L1. IL-10 and IL-17 secretion in the supernatant of B cells was evaluated by ELISA. Data showed that *T. cruzi* stimulation diminished the expression of CD24 and CD38 on CD27^−^ B cells while reducing the percentage of CD24^high^ inside CD27^+^ B cells. Furthermore, *T. cruzi* induced a regulatory B cell phenotype by increasing B10 cells and IL-10 secretion in all the groups. The innate-like B10 cells expansion observed in patients with cardiomyopathy would be associated with CD27^−^ B10 cell subsets, while no predominant phenotype was found in the other groups. Patients with cardiomyopathy also displayed higher IL-17 secretion levels in *T. cruzi*–activated B cells. CpG + CD40L stimulation revealed that B cells from CCD patients and NI donors had the same ability to differentiate into B10 cells and secrete IL-10 *in vitro*. Additionally, CCD patients showed an increased frequency of CD24^−^CD27^−^ B cells and a reduction in the percentage of CD24^high^CD27^+^ Breg cells, which appeared to be inversely correlated with the presence of *T. cruzi* DNA in blood. Finally, CCD patients exhibited a higher frequency of PD-L1^+^ B cells in *T. cruzi*–stimulated samples, suggesting that IL-10-independent mechanisms could also be tangled in the control of inflammation. Altogether, our results provide evidence about the potential role of Breg cells in the immune response developed against *T. cruzi* and its contribution to chronic Chagas cardiomyopathy.

## Introduction

Chagas disease, which etiological agent is the parasite *Trypanosoma cruzi*, remains affecting about 6 to 7 million people worldwide, mostly in Latin America, but also in the United States of America, Canada, and many European and some African, Eastern Mediterranean, and Western Pacific countries (WHO, 2021). After infection with the parasite, the innate and adaptive immunity elicited by the host leads to rapid control of the acute parasitemia (Cardoso et al., 2016). However, if the infected individuals have no access to the current available antiparasitic drug, Benznidazol, the infection cannot be completely resolved, and the disease progresses to the chronic phase. The clinical features of the chronic Chagas disease (CCD) vary widely. About 70% of patients experience no detectable signs or symptoms and can stay symptom-free for years or even for the entirety of their lifetime, while up to 30% of patients develop cardiac alterations and up to 10% suffer from digestive lesions (particularly enlargement of the esophagus or colon) or both manifestations (cardiac and digestive) (Pérez-Molina and Molina, 2018). The immune response, encompassing all its stimulatory and regulatory mediators, is one of the main factors that tilt the balance between the two clinical forms of CCD (Acevedo et al., 2018; Chevillard et al., 2018).

Immature transitional CD19^+^CD24^high^CD38^high^ and CD19^+^CD24^high^CD27^+^ B cells have immunosuppressive capacity and are considered the most representative subsets of the so-called regulatory B (Breg) cells in human peripheral blood (de Masson et al., 2014; Oleinika et al., 2018). CD19^+^CD24^high^CD38^high^ B cells inhibit the differentiation of CD4^+^ T cells into the pro-inflammatory Th1 and Th17 and promote Treg cells (Blair et al., 2010; Flores-Borja et al., 2013). Deficiencies in the number and function of these cells have been first reported in patients with systemic lupus erythematosus (Blair et al., 2010), spreading then to other autoimmune diseases (multiple sclerosis, rheumatoid arthritis, Sjögren’s syndrome), allergic rhinitis, and chronic infections (Abebe et al., 2021). Furthermore, CD19^+^CD24^high^CD38^high^ Breg represents a high percentage of the bulk of B lymphocyte in cord and peripheral blood of healthy neonate and was found increased in late-onset sepsis, linking these cells with pregnancy success (Li et al., 2018). On the other hand, CD19^+^CD24^high^CD27^+^ B cell subset harnesses its immunomodulatory activity mostly by regulating the production of pro-inflammatory cytokines, tumor necrosis factor (TNF)-α in monocytes and interferon (IFN)-γ in CD4^+^ T cells (Iwata et al., 2011; Hasan et al., 2019; Jansen et al., 2021). Alterations in the frequency of this memory-like Breg have been detected in similar scenarios than the CD19^+^CD24^high^CD38^high^ Breg, including cancer (Abebe et al., 2021; Jansen et al., 2021).

However, and depending on the different disease models, stimulation conditions as well as the panel of cell surface markers used in the flow cytometry analysis, other B cells are also classified as Breg. Thus, CD19^+^CD5^+^CD1d^+^ (Zhang et al., 2012; Zhang et al., 2014), CD19^+^Tim-1^+^ (Aravena et al., 2017; Gu et al., 2017b), and CD19^+^CD25^+^CD71^+^CD73^−^ cells (van de Veen et al., 2013) participate by ameliorating the inflammatory response elicited in different immune-related pathologies (Abebe et al., 2021). Although still under investigation, the immunomodulatory mechanisms exerted by these heterogeneous mix of B cells encompass mainly the secretion of the anti-inflammatory cytokine interleukin (IL)-10, but also IL-35 and transforming growth factor (TGF)-β production, or the expression of the surface molecules, like CD80, CD86, Fas ligand (FasL), programmed death ligand-1 (PD-L1), CD40L, or CD1d, *via* direct cell–cell interactions (Harris et al., 2000; Rosser and Mauri, 2015; Abebe et al., 2021).

Data on the role of Breg cells in chronic *T. cruzi* infection in humans are scarce. In a first study, Fares et al. (2013) evaluated the expression of CD5 and CD1d markers and IL-10 production within CD19^+^ B cells in an *ex vivo* context. The authors observed that IL-10 production in CD19^+^CD5^+^CD1d^+^ B cells is higher in patients with CCD when compared with non-infected individuals. But remarkably, they also found that the percentage of IL-10 within CD19^+^CD5^−^CD1d^−^ subsets is increased in patients with the cardiac form of CCD, facing the conundrum about the other phenotype of B cells with regulatory functions (Fares et al., 2013). Recently, we demonstrated, in an *ex vivo* study and focusing on CD24, CD27, and CD38 cell surface markers, that the phenotypic profile of B cells is altered in patients with CCD, depending on their clinical status (Girard et al., 2021). Hence, total B cells and transitional CD24^high^CD38^high^ B cell subpopulation are expanded in CCD patients with cardiac involvement compared to non-infected donors, while the CD24^high^CD27^+^ B cell subset remains unchanged. In addition, and although no differences were observed in the frequency of total IL-10-producing B cells (B10) between patients with and without cardiac alterations, the former showed an increased proportion of CD24^int^CD38^int^ naïve B10 cells and a trend to a lower frequency of CD24^high^CD38^low^ memory B10 cells. Since naïve and memory B10 cells have different functions in immune-related diseases, we speculate that naïve B10 cells would be involved in the maintenance of the autoimmune disorders raised in cardiac patients. In the same line of finding, we observed that the phenotypic distribution within B10 cells in terms of naïve, transitional, and memory subsets in CCD patients without cardiac involvement displays the same pattern detected in non-infected individuals.

In the present study, we have expanded on those previous results by broadly delving the phenotypic traits of B and B10 cells from CCD patients with or without cardiac alterations and non-infected donors, in response to *T. cruzi* stimulation. For this purpose, isolated B cells were examined by flow cytometry using the combination of CD19, CD24, CD27, and CD38 cell surface markers, while the secretion of IL-10 and IL-17 was analyzed in the culture supernatant by ELISA. It was also our interest to phenotypically characterize Breg cells under the non-specific stimulation with cytosine-phosphate-guanosine (CpG) oligodeoxynucleotide plus CD40L. Finally, the expression of PD-L1 was explored on B cells as another mediator, in addition to IL-10, for Breg cells function in the context of human chronic Chagas disease.

## Materials and Methods

### Study Population and Blood Sample Collection

Donors were enrolled either at Instituto Nacional de Parasitología “Dr. Mario Fatala Chabén” or at Hospital General de Agudos “Dr. Ignacio Pirovano,” Buenos Aires, Argentina. *T. cruzi* infection was diagnosed according to national and international guidelines by indirect immunofluorescence assay (IFI), indirect hemagglutination assay (IHA), and enzyme-linked immunosorbent assay (ELISA) (WHO, 2002). Subjects positive in at least two of these tests were considered to be infected. Patients in the chronic phase of the infection underwent a complete clinical and cardiological examination and were grouped according to a modified version of the Kuschnir classification (Ribeiro et al., 2012) as follows: Group 0, patients without demonstrable cardiac pathology (G0, n=13); and Group 1, patients with cardiac involvement (G1, n=13). Subjects in Group 0 (Kuschnir 0 or K0) had a normal ECG and normal chest radiography, whereas individuals within Group 1 had cardiac alterations such as right and/or left branch blockage and different degrees of conductive functional alterations (Kuschnir 1 or K1, n=12) or heart failure symptoms (Kuschnir 3 or K3, n=1). Due to sample availability reasons, most of the donors included in the G1 group belonged to Kuschnir stage 1. A random group of subjects with negative serological tests for *T. cruzi* infection were included as the non-infected control group (NI, n=13). The exclusion criteria included record of history of treatment with Benznidazole or Nifurtimox and presence of systemic arterial hypertension, diabetes mellitus, thyroid dysfunction, renal insufficiency, chronic obstructive pulmonary disease, hydroelectrolytic disorders, alcoholism, history suggesting coronary artery obstruction and/or rheumatic disease, as well as the impossibility of undergoing the examinations. The clinical features of the study population are summarized in **Table 1**. Samples from patients and non-infected donors consisted of 50–60 ml of peripheral venous blood, collected in tubes with EDTA as anticoagulant (Vacutainer, BD Biosciences, NJ, USA). All participants were permanent residents of the greater Buenos Aires area, a location free of *Triatoma infestans*, for at least the last 10 years.

**Table 1:** Demographic and clinical characteristics of donors.

Characteristic	NI (Non-*T. cruzi* infected, n=13)	G0 (patients without cardiac involvement, n=13)	G1 (patients with cardiomyopathy, n=13)
**Age, median (interquartile range)**	42 (32–51)	54 (49–63)	56 (46–62)
**Gender, F/M**	6/7	7/6	7/6
**Kuschnir stage, 0-1-2-3**	NA	13-0-0-0	0-12-0-1
**PCR, +/-**	NA	6/7	5/8
**Anti-*T. cruzi* IgG titer, median (interquartile range)**	<10	2,135 (751–12,217)	6,084 (3,786–13,884)

The titer of anti-T. cruzi antibodies was considered as the maximum reactivity 50 (RM_50_) of the plasma dilution factor. NA, not applicable.

Blood samples were drawn after the purposes of the investigation were explained, and written informed consent was signed from all participants.

### PBMC and Plasma Isolation

An aliquot of 2 ml of whole blood from each participant was separated for parasite load detection, while a similar volume was centrifuged for 15 min at 800 g to separate plasma, which was stored at −20°C for *T. cruzi* specific antibody analysis. The remaining whole blood underwent Ficoll-Hypaque density gradient centrifugation (GE Healthcare Bio-Sciences AB, Uppsala, Sweden), according to manufacturer-provided instructions to obtain PBMC; this procedure was carried out within 4 h of blood collection. Isolated PBMCs were resuspended in heat-inactivated fetal bovine serum (FBS, Natocor, Córdoba, Argentina) containing 10% dimethylsulfoxide (DMSO) and cryopreserved in multiple aliquots in liquid nitrogen until used. After thawing, obtained PBMCs with viability greater than 85% assessed by light microscopy and trypan blue staining were used in the experiments.

### Characterization of Blood Samples

The titer of *T. cruzi*–specific IgG antibodies in the plasma of patients was determined as previously described (Longhi et al., 2014; Girard et al., 2018). For parasite load detection, total DNA was extracted from whole blood aliquots using the High Pure PCR Template Preparation kit (Roche Diagnostics Corp., IN, USA) following manufacturer-provided instructions, and a multiplex real-time PCR was performed as previously described (Duffy et al., 2013; Ramírez et al., 2015).

### *Trypanosoma cruzi* Antigen Preparation

Whole trypomastigote lysate was obtained from *T. cruzi* CL Brener (DTU Tc VI) infected VERO cells (multiplicity of infection, MOI 10:1) supernatants as previously described (Acevedo et al., 2017). Briefly, VERO cells were infected in MEM culture medium supplemented with 10% inactivated FBS, 100 U/ml penicillin, 100 µg/ml streptomycin, and 2 mM l-glutamine (all from Gibco, Thermo Fisher Scientific, Waltham, USA). After 1 h of incubation, extracellular parasites were removed by aspirating cell culture medium followed by the addition of fresh MEM with 3% FBS. Trypomastigotes were harvested from day 6 to 7 post-infection in the supernatant of VERO cells by centrifugation at 2,000 g for 10 min and after cellular debris was removed. Trypomastigotes were washed once with phosphate-buffered saline (PBS) and stored as dry pellet at −80˚C until lysate preparation. Successive parasite collection rounds were performed until obtaining a total of 2.10^9^ total trypomastigotes. To prepare whole antigenic lysate, parasites were resuspended in lysis buffer (1 mM EDTA, 0.1% Tritón X-100, 0.1% NP40, 100 mM NaCl, 1 mM PMSF in distilled water) and submitted to five cycles of freezing-thawing in liquid nitrogen, which were followed by sonication. After lysis procedure, the suspension was filter sterilized through a 0.2-mm-pore-size membrane, assayed for protein concentration, aliquoted, and stored at −80°C until use.

### B Cell Isolation and Culture

B cells were isolated from thawed PBMC by negative selection using magnetic-bead purification MACS kit (EasySep Human Pan B cell isolation kit; Miltenyi Biotec, Bergisch Gladbach, Germany) and the EasySep Purple Magnet (STEMCELL Technologies, Vancouver, Canada), following the manufacturer-provided protocol. The frequency of CD3^−^CD19^+^ cells prior and after magnetic separation was evaluated by FACS staining on a BD FACSCanto II cytometer (BD Biosciences, Franklin Lakes, NJ, USA). The purity of isolated B cells was 91.65 ± 5.58% (mean ± standard deviation). All cultures were performed in RPMI-1640 medium supplemented with 100 U/ml penicillin, 100 µg/ml streptomycin, 2 mM l-glutamine, and 10% FBS. B cells, seeded in 96-well U-bottom plates at a density of 400.000 cells/well, were incubated for 48 h at 37°C in a humidified 5% CO_2_ incubator, with *T. cruzi* trypomastigote lysate (10 µg/ml), CpG oligodeoxynucleotide (2.5 μg/ml; CpG-ODN2006-5’-tcgtcgttttgtcgttttgtcgtt-3’; InvivoGen, San Diego, CA, USA) + recombinant human CD40L (1 μg/ml; Biolegend, San Diego, USA), a combination of *T. cruzi* lysate + CpG + CD40L or culture medium alone as non-stimulated control. For intracellular IL-10 detection, 2 µl/ml of cell stimulation cocktail (eBiosciences, Thermo Fisher Scientific, Waltham, MA, USA) and 1 µl/ml of Brefeldin A (equivalent to 5 µg/ml; Biolegend), formally called PIB, were added for the last 5 h of incubation. Of note, cell stimulation cocktail contains Phorbol-12-myristate-13-acetate (PMA; 0.05 µg/ml) and ionomycin (Iono; 1 µg/ml).

### Flow Cytometry Staining

B cells were transferred to a 96-well V-bottom plate and washed once with PBS by centrifugation at 700 g for 3 min at room temperature (RT). Supernatants were discarded, and cells were incubated for 30 min with a staining solution containing BV421 anti-CD3 (clone UCHT1), PECy5 anti-CD19 (clone HIB19), APC anti-CD27 (clone M-T271), PECy7 anti-CD24 (clone ML5), APCCy7 anti-CD38 (clone HB-7), and FITC anti-PD-L1 (clone MIH1) fluorochrome-conjugated antibodies (Abs) or isotype control Abs specific for human. After surface markers staining, cells were washed with PBS, fixed with Fixation Buffer (Biolegend) for 20 min at RT in the dark, and washed with PBS by centrifugation. For intracellular IL-10 detection, cells were permeabilized with Perm-Wash Buffer (Biolegend), stained with PE anti-IL-10 antibody (clone JES3-9D7) and fixed again for 20 min with Fixation Buffer. Zombie-Aqua Fixable viability dye (Biolegend) was used to exclude non-viable cell events. Fluorescence-minus-one (FMO) controls were used to determine the cut point for the IL-10 staining, and isotype-matched Ab control staining was considered for cell surface markers gate setting. All Abs were purchased from Biolegend and BD Biosciences and were used at optimal concentrations determined by previous titration experiments. A minimum of 20.000 events within the B cell population were acquired in a FACSCanto II flow cytometer using FACS Diva Software (BD Biosciences). Data analysis was performed with FlowJo Software V10 (FlowJo LLC, Ashland, OR, USA). The general gating strategy is illustrated in **Supplementary Figure 1**.

### Cytokine Secretion

IL-10 and IL-17 secretion was measured in culture supernatants of B cells by ELISA (ELISA Max Standard Set Human IL-10, Biolegend and Human IL-17A ELISA Ready-SET-Go!, eBiosciences, San Diego, CA, USA), according to the manufacturer’s instructions. The supernatants were collected after 43 h of culture and before PIB addition and stored at −80°C until the ELISA assay was performed.

### Statistical Analysis

A linear mixed-effects regression model (LMER) was applied to analyze differences in the frequency of cell populations, the mean fluorescence intensity (MFI) of markers, and the cytokine concentration among different stimulation conditions and groups. Group and culture conditions were considered fixed factors, sample was included as random factor, while the frequency of cells and the MFI or cytokine concentration were set as dependent variables. For *post-hoc* comparisons, Tukey HSD (honest significant difference) contrasts were used. The frequency of cells, MFI, and cytokine concentration data were tested for normality and homoscedasticity using Shapiro-Wilk and Bartlett tests, respectively. LMER models were fitted in R version 4.0.2 (R Core Team, 2013) with lmer function from lme4 package (Bates et al., 2010) and glht function from multcomp package (Hothorn et al., 2014). *p-*value less than 0.05 was considered statistically significant.

## Results

### The Phenotype of Breg Cells Changes Upon *In Vitro* Stimulation With *T. cruzi* in Patients With Chronic Chagas Disease

**Figure 1:**
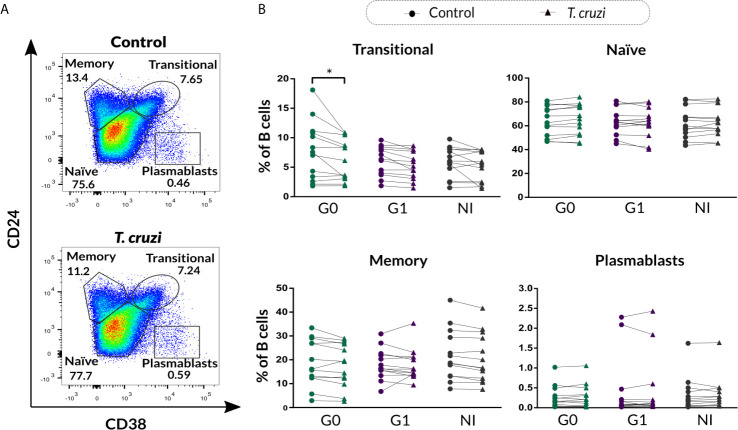
Phenotypic distribution based on CD24 and CD38 expression of isolated B cells from patients with CCD and NI donors upon *T. cruzi* stimulation. **(A)** Gating strategy used to identify B cell subsets according to CD24 and CD38 expression. Dot plots from one NI donor are illustrated. **(B)** Frequency of transitional, naïve, memory, and plasmablasts subsets in Control or *T. cruzi* stimulated B cells from CCD patients (G0, G1) and NI donors. Each symbol represents data from one individual. Statistically significant differences are indicated with an asterisk (*) *p *< 0.05.

Aiming to characterize Breg cell compartment in patients with CCD with different clinical forms and NI donors following *T. cruzi* stimulation, B cells isolated from PBMC were incubated with *T. cruzi* lysate or medium only (control) during 48 h with the addition of PIB in the last 5 h of culture, and then applied flow cytometry to evaluate the expression of CD24, CD27, and CD38 cell surface markers. By using the combination of CD24 and CD38, we assessed the well-established subpopulations of B cells, identified as CD24^high^CD38^high^ (immature transitional), CD24^int^CD38^int^ (primarily mature naïve), CD24^high^CD38^low^ (primarily memory), and CD24^low^CD38^high^ (plasmablasts) (**Figure 1A**). Results showed that *T. cruzi* lysate stimulation induced a decrease in the frequency of immature transitional B cells in patients without cardiac involvement (*p*=0.002), although no statistically significant differences were found among the groups (**Figure 1B**). The rest of the subpopulations stayed unchanged under these conditions and was comparable among the groups (**Figure 1B**).

**Figure 2:**
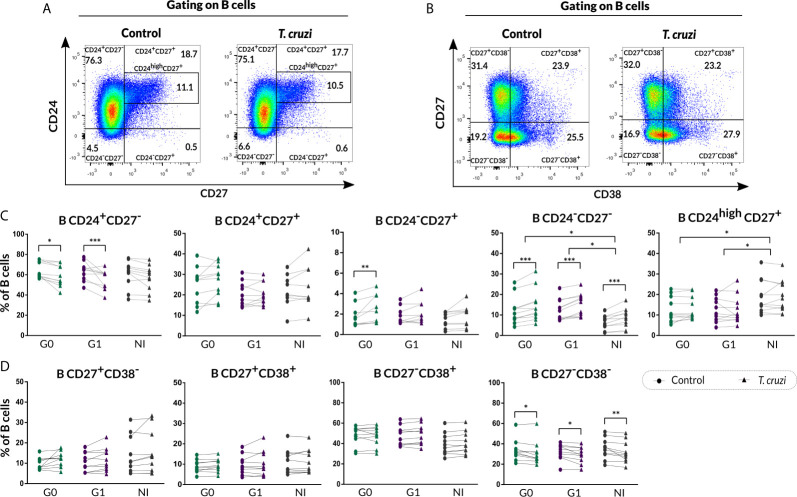
Phenotypic distribution based on CD24-CD27 and CD27-CD38 markers expression of isolated B cells from CCD patients and NI donors upon *T. cruzi* stimulation. **(A, B)** Dot plots showing the gating strategy used to identify B cell subsets defined by CD24-CD27 and CD27-CD38 expression, respectively. **(C, D)** Frequencies of B cell subpopulations in CCD patients (G0, G1) and NI donors in Control or *T. cruzi*–stimulated samples, according to CD24-CD27 and CD27-CD38 markers expression, respectively. Each symbol represents data from one individual. Statistically significant differences are indicated with asterisks (*) *p* < 0.05, (**) *p* < 0.01, (***) *p* < 0.001.

We further evaluated the percentage of B cell subsets according to CD24-CD27 (**Figure 2A**) and CD27-CD38 (**Figure 2B**) markers expression upon *T. cruzi* stimulation. Considering the former combined expression, patients with CCD with and without cardiac involvement showed a minor frequency of CD24^+^CD27^−^ B cells in *T. cruzi*–stimulated cultures, compared to NI donors (G0: *p*=0.025, G1: *p*<0.001; **Figure 2C**). The frequency of CD24^−^CD27^−^ subset increased in response to *T. cruzi* antigens in subjects from the three groups (*p<*0.001), whereas only patients without cardiac involvement showed an expansion of CD24^−^CD27^+^ B cell subset (*p*=0.0037, **Figure 2C**). Interestingly, the percentage of CD24^high^CD27^+^ B cells was lower in G0 and G1 groups (G0 *vs.* NI: *p*=0.02, G1 *vs.* NI: *p*=0.014; **Figure 2C**), and the magnitude of this subset was found diminished in those patients with detectable *T. cruzi* DNA in peripheral blood (PCR^+^), independently of their clinical form (*p*=0.007). Conversely, CD24^−^CD27^−^ B cell frequency was higher in G0 and G1 groups (G0 *vs.* NI: *p*=0.032, G1 *vs.* NI *p*=0.02; **Figure 2C**). Of note, CD24^−^CD27^−^ and CD24^high^CD27^+^ B cell alterations in CCD patients were observed independently of stimulation condition (**Figure 2C**). Concerning the percentage of CD24^+^CD27^+^ B cell subset, no statistically significant differences were detected neither upon *T. cruzi* stimulation nor comparing among the groups (**Figure 2C** **)**. Then, we examined the frequencies of B cell subsets based on CD27-CD38 expression. A reduced frequency of the double negative population (CD27^−^CD38^−^) was identified in *T. cruzi*–stimulated B cells from patients with CCD and NI donors (G0: *p*=0.049, G1: *p*=0.011, NI: *p*=0.0044), with no differences among the groups (**Figure 2D** **)**. Besides, the percentages of CD27^+^CD38^−^, CD27^+^CD38^+^, and CD27^−^CD38^+^ B cell subsets did not change after *in vitro* stimulation with *T. cruzi*, and no statistically significant differences were detected by comparing the groups (**Figure 2D**).

The alterations of Breg cells unveiled upon unspecific stimulation in patients with some autoimmune diseases and cardiomyopathies (Bankó et al., 2017; Jiao et al., 2018) led us to examine the phenotypic distribution of B cells after stimulation with the oligonucleotide CpG plus recombinant CD40L (CpG + CD40L) in our cohort of CCD patients and NI donors (**Supplementary Figures 2A, B**, **3A–D**). The change induced by CpG + CD40L stimulus was almost equivalent all across the B cell subpopulations in the three groups of subjects; an increase of plasmablasts and a reduction of CD24^high^CD27^+^ subpopulation were detected in NI donors (**Supplementary Figures 2B, 3C**). Furthermore, the most substantial differences were found in the augmentation of CD24^+^CD27^+^ in G0 and NI, together with a drop in the frequency of and CD27^−^CD38^+^ B cells in CCD patients with cardiomyopathy (**Supplementary Figures 3C, D**).

### *Trypanosoma cruzi* Induces an Innate-Like Regulatory B10-Cell Phenotype in CCD Patients With Different Clinical Forms and NI Donors

**Figure 3:**
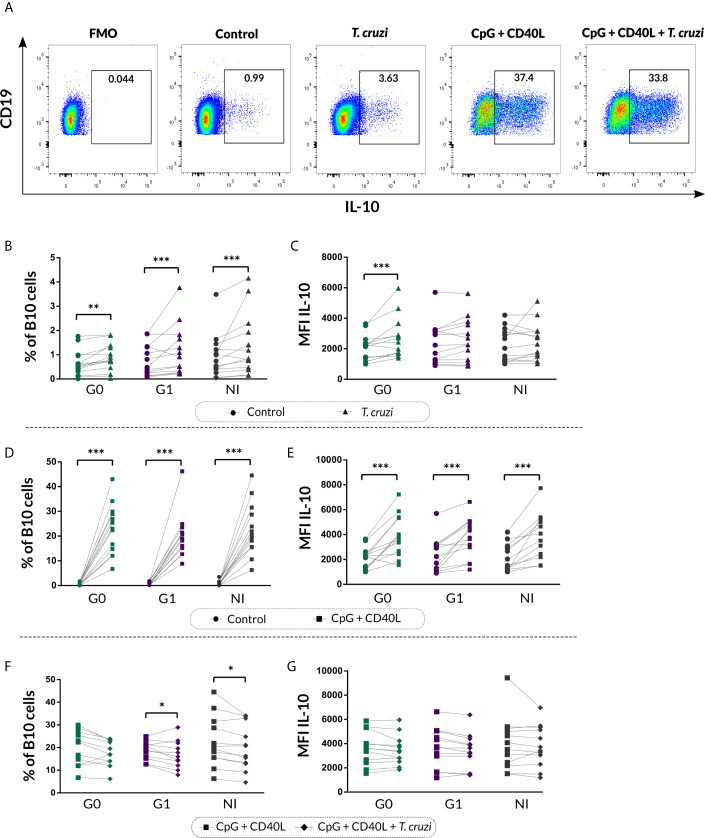
Frequency of B10 cells and MFI of IL-10 on isolated B cells from patients with CCD and NI donors upon stimulation with *T. cruzi* or CpG + CD40L. **(A)** Dot plots from one NI donor showing the gating strategy used to determine the frequency of total B10 cells on B cell cultures following different stimulus. Fluorescence-minus-one (FMO) controls for each donor were used to determine the cutoff for IL-10 staining on B cells. **(B, C)** Frequency of B10 cells and MFI for IL-10 on Control B cells or *T. cruzi–*stimulated samples. **(D, E)** Frequency of B10 cells and MFI for IL-10 on Control B cells or CpG + CD40L stimulated samples. **(F, G)** Frequency of B10 cells and MFI for IL-10 on B cells stimulated with CpG + CD40L or CpG + CD40L + *T. cruzi*. Each symbol represents data from one individual. Statistically significant differences are indicated with asterisks (*) p < 0.05, (**) p < 0.01, (***) p < 0.001.

We next characterized the B10 cell population from isolated B cell stimulated with *T. cruzi* or with the B10 cell differentiation-inducing molecules CpG + CD40L, in CCD patients and NI donors. As was illustrated in **Figure 3A**, FMO controls were used to determine IL-10^+^ B cells in each sample. This analysis revealed that *T. cruzi* stimulation increased total B10-cell frequency in both groups of CCD patients and NI donors (*p*<0.001; **Figure 3B**), while no statistical differences were found among the groups. Interestingly, the mean fluorescence intensity (MFI) for IL-10 in B10 cells significantly raised in patients without cardiac involvement (*p*<0.001; **Figure 3C**). In parallel, CpG + CD40L stimulation induced a great expansion not only in the frequency of B10 cells but also in MFI compared with culture medium only (*p*<0.0001; **Figures 3D, E**), suggesting that B cells from patients with CCD and NI donors have the same ability to differentiate into B10 cells *in vitro* (**Figures 3D, E**).

We therefore asked whether *T. cruzi* could alter the B cell activation exerted by the non-specific stimulus. To address this question, B10 cell frequency and MFI for IL-10 were evaluated in B cells incubated with CpG + CD40L + *T. cruzi* and compared with CpG + CD40L–stimulated samples. Results showed that the percentage of B10 cell diminished in CpG + CD40L + *T. cruzi*–stimulated B cells in patients with cardiac involvement and NI donors (G1: *p*=0.024, NI: *p*=0.017; **Figure 3F**), although no differences were detected in patients belonging to G0. IL-10 MFI levels were comparable between both conditions and also among the groups (**Figure 3G**).

**Figure 4:**
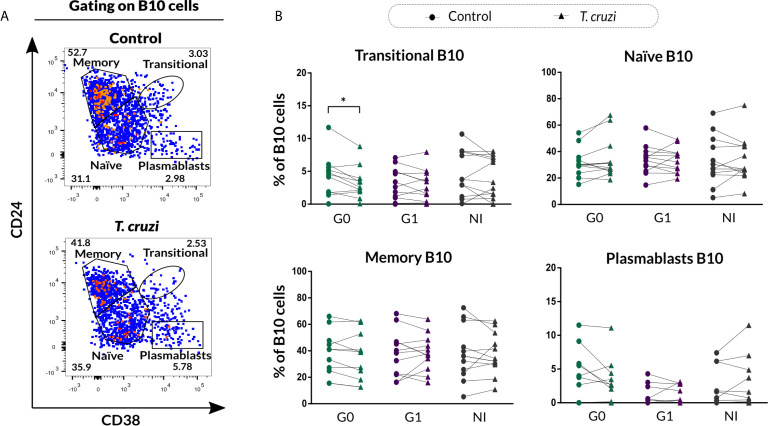
Phenotypic distribution of B10 cells based on CD24 and CD38 expression from patients with CCD and NI donors upon *T. cruzi* stimulation. **(A)** Dot plots showing the gating strategy used to identify B10 cell subsets according to CD24 and CD38 expression for one NI donor. Large dots were used to facilitate visibility of events. **(B)** Frequency of transitional, naïve, memory, and plasmablasts B10 cell subsets in Control or *T. cruzi* stimulated B cells from CCD patients (G0, G1) and NI donors. Each symbol represents data from one individual. Statistically significant differences are indicated with an asterisk (*) p < 0.05.

In order to investigate the phenotypic distribution of B10 cells based on CD24 and CD38 markers, the gating strategy shown in **Figure 4A** was applied to analyze the IL-10 secreting B cells within transitional, naïve, memory, and plasmablast subsets in control and *T. cruzi*–stimulated cultures. A significant drop in the percentage of transitional B10 cells (IL-10^+^CD24^high^CD38^high^) was detected only in subjects of the G0 group (*p*=0.010), and no differences were detected among the groups (**Figure 4B**). With regard to naïve, memory, and plasmablasts B10 subsets frequency, no statistically significant differences were found in response to *T. cruzi* stimulation (**Figure 4B**).

**Figure 5:**
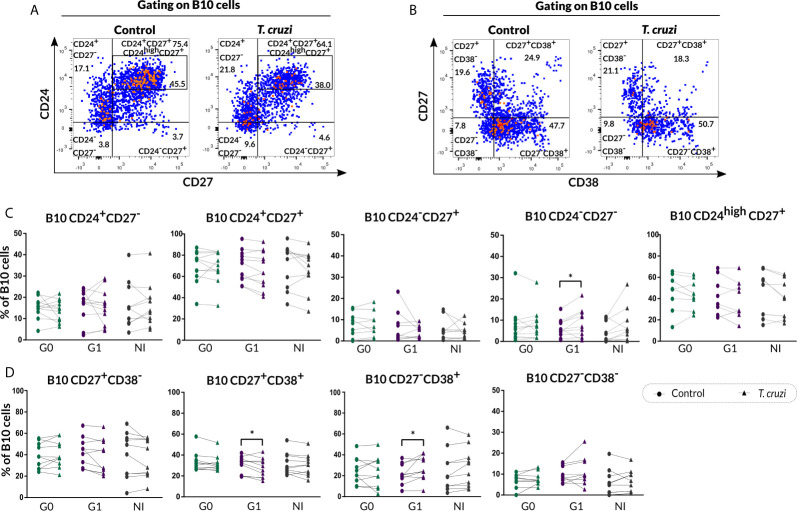
Phenotypic distribution of B10 cells in terms of CD24-CD27 and CD27-CD38 markers expression from CCD patients and NI donors upon *T. cruzi* stimulation. **(A, B)** Dot plots corresponding to one NI donor showing the gating strategy used to identify B cell subsets defined by CD24-CD27 and CD27-CD38 expression, respectively. **(C, D)** Frequencies of B cell subpopulations from CCD patients (G0, G1) and NI donors in Control or *T. cruzi*–stimulated samples, according to CD24-CD27 and CD27-CD38 markers expression, respectively. Each symbol represents data from one individual. Statistically significant differences are indicated with asterisks (*) *p * < 0.05.

The phenotypic traits of B10 cells were also evaluated according to CD24-CD27 (**Figure 5A**) and CD27-CD38 (**Figure 5B**) markers expression in *T. cruzi*–stimulated B cells. The analysis showed a higher frequency of CD24^−^CD27^−^ B10 cells in patients with cardiac involvement (*p*=0.013), while no additional differences were identified in the other phenotypic CD24-CD27 subpopulations (**Figure 5C** **)**. Furthermore, patients of the G1 group displayed a diminished percentage of CD27^+^CD38^+^ B10 subset (*p*=0.027) and an augmented percentage of CD27^−^CD38^+^ B10 subset (*p*=0.023) in *T. cruzi*–stimulated samples (**Figure 5D**). Additionally, CD38 negative B10 cell subsets were not altered between conditions and neither among the groups (**Figure 5D**).

Similar to the analysis carried out in B cells, we sought to study B10 cell subsets upon CpG + CD40L stimulation (**Supplementary Figures 4A, B, 5A–D**). Our results showed comparable changes in the frequencies of the distinct phenotypes except for the CD27^−^CD38^+^ subpopulation, which was augmented in CCD patients with no significant differences in NI group donors (**Supplementary Figure 5D**).

### The Secretion of IL-17 Increases in B Cells From Patients With the Cardiac Form of CCD

**Figure 6:**
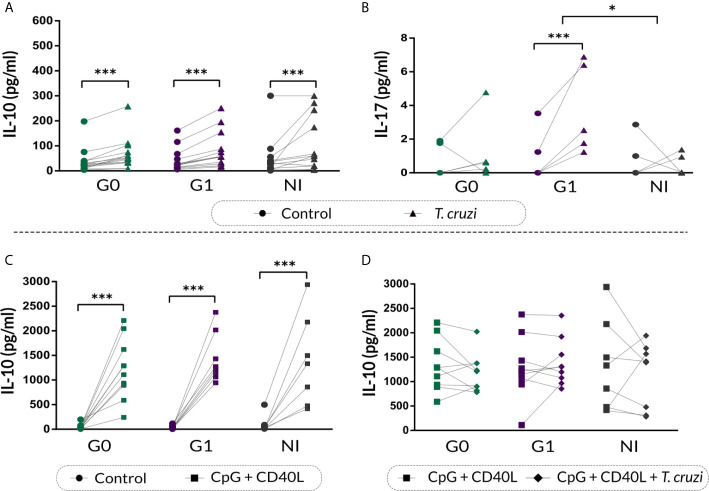
IL-10 and IL-17 cytokine secretion on supernatant of B cells from patients with CCD and NI donors following *T. cruzi* or CpG + CD40L stimulation. **(A, B)** IL-10 and IL-17 secretion in supernatants of Control or *T. cruzi*–stimulated B cells from CCD patients (G0, G1) and NI donors. **(C)** IL-10 secretion in supernatants of Control or CpG + CD40L stimulated B cells from CCD patients and NI donors. **(D)** IL-10 secretion in supernatants of B cells following stimulation with CpG + CD40L or CpG + CD40L + *T. cruzi* in CCD patients and NI donors. Each symbol represents data from one individual. Statistically significant differences are indicated with asterisks (*) *p* < 0.05, (***) p < 0.001.

IL-10 and IL-17 secretion in the supernatants of stimulated B cell cultures was quantified by ELISA. Results showed that *T. cruzi* increased IL-10 secretion in CCD patients and NI donors (*p*<0.001; **Figure 6A**), and no differences were found among the groups. Moreover, patients with the cardiac form exhibited higher IL-17 secretion in the supernatant of *T. cruzi*–stimulated B cells in contrast to controls (*p*<0.001), while patients without cardiac involvement and NI donors did not show statistically significant differences (**Figure 6B**). When comparing among the groups, statistically significant differences were found only between patients with cardiomyopathy and NI individuals (*p*=0.031; **Figure 6B**).

We further assessed IL-10 secretion in the supernatant of B cells incubated with CpG + CD40L, and similar increased levels were detected in all the groups (*p*<0.001; **Figure 6C**). However, this augmentation was slightly higher in patients with cardiac involvement (mean percentage of increase, G0: 2930%, G1: 4340%, NI: 1533%). On the other hand, none of the groups showed changes in IL-10 secretion upon CpG + CD40L + *T. cruzi* stimulation compared with CpG + CD40L (**Figure 6D**).

### B Cells From Patients With CCD Exhibit Higher Levels of the Regulatory Molecule PD-L1 Upon *T. cruzi* Stimulation

**Figure 7:**
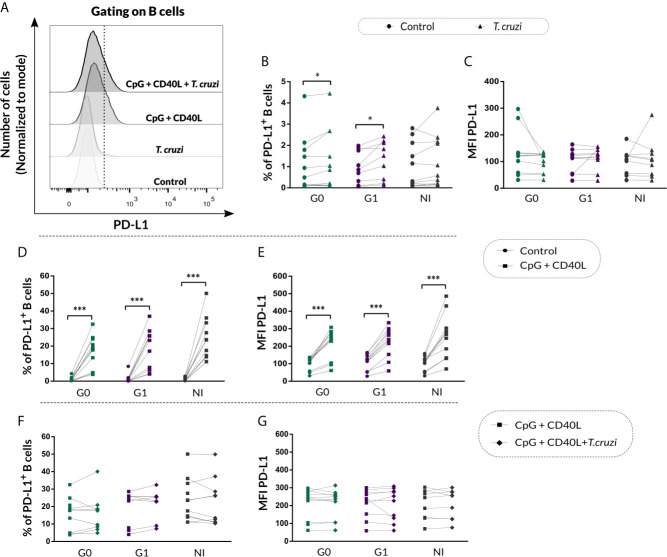
PD-L1 expression on isolated B cells from patients with CCD and NI donors following *T. cruzi* or CpG + CD40L stimulation. **(A)** Fluorescence histograms showing PD-L1 expression in *T. cruzi*, CpG + CD40L, or CpG + CD40L + *T. cruzi*–stimulated or Control B cells. Data from one NI donor are illustrated. Dotted line shows the cut point to identify PD-L1^+^ B cells (CD19^+^PD-L1^+^). **(B, C)** Frequency of PD-L1^+^ B cells and MFI for PD-L1 on Control or *T. cruzi*–stimulated B cells from CCD patients (G0, G1) and NI donors. **(D, E)** Frequency of PD-L1^+^ B cells and MFI for PD-L1 on Control or CpG + CD40L–stimulated B cells from CCD patients (G0, G1) and NI donors. **(F, G)** Frequency of PD-L1^+^ B cells and MFI for PD-L1 on CpG + CD40L or CpG + CD40L + *T. cruzi*–stimulated B cells from CCD patients (G0, G1) and NI donors. Each symbol represents data from one individual. Statistically significant differences are indicated with asterisks (*) *p* < 0.05, (***) p < 0.001.

Given that B cells can modulate the immune response through IL-10-independent mechanisms, such as PD-L1 regulatory molecule expression (Khan et al., 2015), we decided to measure PD-L1 expression on B cells from CCD patients and NI donors following the same stimulation conditions mentioned above. In **Figure 7A**, fluorescence histograms from one representative NI donor show the analysis strategy used to evaluate PD-L1 expression on B cells in control cultures or upon *T. cruzi*, CpG + CD40L, and CpG + CD40L + *T. cruzi* stimulation. Results evidenced an increase in the percentage of PD-L1^+^ B cells in CCD patients with different clinical forms upon *T. cruzi* stimulation (G0: *p*=0.032, G1: *p*=0.0029; **Figure 7B**), but no differences were detected in MFI for PD-L1 on B cells (**Figure 7C**).

In addition, both, the frequency of PD-L1^+^ B cells (*p*<0.001; **Figure 7D**) and MFI for this marker (*p*<0.0001; **Figure 7E**) increased in response to the non-specific stimulation with CpG + CD40L in CCD patients and NI donors, and this augmentation remained unchanged in the presence of *T. cruzi* lysate for all the groups (**Figures 7F, G**).

## Discussion

The balance between effector and regulatory functions of the different immune cells is pivotal for maintaining a homeostatic state both in health and in an ongoing infection. Alterations in the phenotype or function of Breg cells contribute to the pathogenesis of immune-mediated diseases. In this work, we evaluated potential phenotypic alterations of Breg cells from patients with CCD with or without cardiomyopathy, as well as from non-infected donors in response to *T. cruzi* stimulation.

The results of our approach showed that *T. cruzi* stimulation alters the expression of surface markers associated with regulatory phenotypes such as CD24, CD27, and CD38 on isolated B cells from patients with CCD and non-infected donors. Hence, *T. cruzi* reduced the expression of CD24 in CD27^−^ B cells in CCD patients and NI donors, and also in CD27^+^ B cell population but only in patients without cardiac manifestations. CD24 is a highly glycosylated protein located in the plasma membrane of B cells, usually as glycosyl-phosphatidylinositol (GPI)-anchored protein or forming part of lipid complexes (lipid rafts) (Mensah et al., 2018). In mature human B cells, the expression of CD24 fluctuates throughout their life cycle until it is lost when they differentiate into plasma cells. Sanz et al. (2008) identified a high expression of CD24 in CD27^+^ B cells, while CD27^−^ presented a low expression of this molecule. In addition, it was shown that CD24 expression level is related to the energy metabolism of B cells throughout their differentiation, and in turn, this role differs among different subpopulations of B cells. *In vitro* assays revealed that CD24^−^ B cells proliferate more actively, while CD24^+^ B cells are more likely to remain in an anergic state or die by apoptosis upon stimulation (Mensah et al., 2018). In face of our results, we hypothesize that parasite antigens would be contributing to the predominance of naive and memory CD24^−^ B cells, more prone to proliferate when stimulated and less able to persist in a state of anergy or undergo apoptosis when faced with signals from the environment. The meaning of this mechanism should be explored in order to understand whether it has a protective or detrimental role in B cell response to *T. cruzi* infection.

Interestingly, an increased frequency of CD24-CD27 double negative B cell subset and a decreased proportion of CD24^high^CD27^+^ B cells were detected in patients with CCD compared with non-infected subjects regardless of the culture condition (control or *T. cruzi* stimulated). A recent study evidenced that CD24^high^CD38^high^ and CD24^high^CD27^+^ populations, contrary to the CD24^−^CD27^−^ B cell subset, can suppress proliferation and secretion of IFN-γ and IL-17 in CD4^+^ T cells. Furthermore, this ability depends on the production of IL-10 and the regulatory molecule PD-L1 (Hasan et al., 2019). In line with our findings, the minor percentage detected of CD24^high^CD27^+^ subset seems to be a trait that was not related with *T. cruzi* stimulation on B cells directly; however, it was associated with CCD. Conversely, the increase in the CD24^−^CD27^−^ subset was directly associated with *T. cruzi* antigen stimulation, since it was found in cells from CCD patients, who already had a higher frequency, and also in B cells from non-infected donors. We could speculate that in chronic *T. cruzi* infection, CD24^−^CD27^−^ B cell subset would be favored in detriment of populations with high expression of both markers, thus contributing to the Breg population being defective in CCD. We also found a negative association between the frequency of the CD24^high^CD27^+^ subset and the level of parasitemia in CCD patients, which suggest that these cells could be related to the control of parasite clearance in the chronic phase of Chagas disease.

CD24^high^CD27^+^ Breg cell population is characterized by a high expression of the suppressive cytokine IL-10, but also by other regulatory molecules such as PD-L1, TGF-β1, Granzyme B, as well as the CD39 ectonucleotidase (Hasan et al., 2019), which is also expressed in Treg cells (Gu et al., 2017a), in suppressor cells of myeloid origin (Li et al., 2017), as well as in other Breg cell populations (Kaku et al., 2014; Figueiró et al., 2016). Several studies have reported that adenosine converted from ATP by the combined activity of CD39 and CD73 ectoenzymes is a newly recognized “immune checkpoint mediator” that suppresses the immune response in various pathologies (Antonioli et al., 2013). In the context of Chagas disease, Santos et al. (2009) demonstrated that one of the mechanisms by which *T. cruzi* can evade the immune response is through the expression of ectonucleotidases of the CD39 family, since inhibitors of this enzyme favor infectivity and virulence of the parasite in *in vitro* and *in vivo* conditions. Even more, a transient increase in CD39/CD73 attenuated the inflammatory macrophage response against *T. cruzi*, and CD73 inhibition was beneficial in establishing the inflammatory profile of macrophages in the myocardium of mice with acute *T. cruzi* infection (Ponce et al., 2016). This finding emphasizes the role of these molecules in modulating the local immune response in the myocardium. Likewise, a recent study showed that Treg cells from patients with the indeterminate form of CCD express higher levels of CD39, indicating that this molecule would be involved in the control of inflammation in these patients (Damasio et al., 2019). In the same way that *T. cruzi* expresses molecules of the CD39 family that modulate the immune response, it could also induce an increase in the expression of these ectonucleotidases on B cells or decrease the subsets that typically express high levels of these enzymes, such as the CD24^high^CD27^+^ B cell population, thus favoring persistence and contributing to disease progression.

By analyzing IL-10-producing B cells, we found that *T. cruzi* increased the frequency of total B10 cells and IL-10 secretion in the culture supernatant from CCD patients and NI subjects. These findings suggest that *T. cruzi* induces a B10 cell regulatory phenotype that does not depend on antigen experience, but constitutes an “innate-like” B cell response to relatively short stimulations (48 h) with parasite antigens. In the same line of thought and given that the experiments were carried out using isolated B cells, IL-10 production would be induced by signaling through a direct engagement of Toll-like receptors (TLR) and/or B cell receptor (BCR) with parasite molecules or through the secretion of autocrine cytokines. It is known that not only TLR agonists such as CpG (TLR9) and R848 (TLR7 and TLR8) are capable of inducing IL-10 production in B cells, but also IFN-α modulates IL-10 production mediated by activation of TLR7 and TLR8. Particularly, Liu et al. (2014) reported that IFN-α regulates the production of IL-10 in B cells through signaling *via* ERK and STAT3. Upon *T. cruzi* infection, the parasite is internalized by macrophages and dendritic cells by different mechanisms, and once inside the phagolysosome, its DNA and RNA contact the TLR7 and TLR9 receptors. This interaction promotes the activation of NF-kβ through the Myd88 signaling pathway (Bartholomeu et al., 2008). As with these other APCs, the activation of TLR7 and TLR9 could occur in B cells by the different antigens contained in *T. cruzi* lysate, promoting the production of IL-10. It is also important to consider that in B cells there is a constant mechanism of cross-talk between TLR7 and TLR9 activation and direct signaling through BCR stimulation. In fact, this joint signaling is essential in the development of self-reactivity mechanisms, promoting or decreasing the activation of potentially harmful B cells (Suthers and Sarantopoulos, 2017). When excessive cellular or tissue damage occurs, the activation of TLR7 or TLR9 by endogenous nucleic acids can be exacerbated, leading to autoimmunity phenomena (Suthers and Sarantopoulos, 2017). In CCD, TLR-BCR signaling mediated by certain *T. cruzi* antigens as well as endogenous antigen products of the immune response itself could also be contributing to the development of autoreactive mechanisms in patients, as well as to activation of potentially harmful or dysfunctional B10 cells.

By reducing the innate and adaptive immune responses, IL-10-mediated suppression can contribute to favorable conditions for the persistence of pathogens and lead to chronic infection. On the other hand, it prevents the immune-mediated tissue lesions as a result of an exacerbated inflammatory state (Mege et al., 2006; Couper et al., 2008). In both the acute and chronic phases of the disease, *T. cruzi* induces an increase of IL-10 as part of its escape mechanism, which makes it difficult to interpret whether the secretion of this cytokine is a cause or a consequence of parasitemia in patients (Flávia Nardy et al., 2015; Cardoso et al., 2016). A recent study showed a positive relationship between the IL-10 levels in sera of CCD patients with detectable parasitemia (PCR^+^) (Salvador et al., 2020). However, in our cohort of patients, we did not find an association between parasitemia and the frequency of B10 cells or IL-10 secretion levels in B cell culture supernatants, suggesting that other cell types are the source of IL-10 in chronic *T. cruzi* infection. In fact, as it was mentioned before, a negative correlation between parasite detection in blood and the percentage of CD24^high^CD27^+^ B cell exists in CCD patients, and this subset is identified as the central IL-10 secreting B cells in humans (Jansen et al., 2021).

The alterations detected in the phenotypic distribution of B10 cells upon stimulation with *T. cruzi* disclose that their increase in patients with Chagas disease cardiomyopathy would be mainly associated with those populations lacking CD27, such as CD24^−^CD27^−^ and CD27^−^CD38^+^. Interestingly, the former was also found augmented in our *ex vivo* analysis (Girard et al., 2021), underpinning this subpopulation as a possible trait in patients with the cardiac form of CCD, and if so, it requires further investigation. Under the same experimental stimulation with CpG + CD40L, Jiao et al. (2018) observed that patients with Idiopathic Dilated Cardiomyopathy (IDC), a heart disease sharing some features with the cardiac form of CCD, presented a decrease in total B10 cells compared to healthy individuals, particularly within CD24^high^CD27^+^ subset. What is even more interesting is that the frequency of B10 cells correlated with parameters of cardiac function in patients, suggesting the contribution of these cells in the severity of heart damage and the progression of IDC (Jiao et al., 2018). Our data proved to be similar between patients with CCD and non-infected donors, illustrating that the Breg response mounted against the stimulus, different from the parasite itself, could be equally effective.

An additional finding of our study was the higher IL-17 secretion by B cells upon *T. cruzi* stimulation in patients with cardiac involvement. Studies *in vitro* with human tonsillar cells pinpoint B cells as an important source of IL-17 (B17 cells) in response to *T. cruzi* infection, although the specific phenotype of these cells has not yet been identified (Bermejo et al., 2013). Likewise, studies evaluating IL-17 in patients infected with *T. cruzi* suggest a protective immunomodulatory function of this cytokine to prevent cardiac damage in CCD (Amezcua Vesely et al., 2020). Patients with cardiac manifestations have lower levels of IL-17 in total lymphocytes and a lower frequency of Th17 cells than patients without clinical symptoms. In turn, IL-17 levels positively correlate with better cardiac function (Magalhães et al., 2013; Sousa et al., 2017). Our results also align with these findings and point to a protective function of B17 cells in patients undergoing heart disease.

Finally, we investigated whether Breg cells could modulate inflammatory responses by mechanisms other than IL-10 production, for example by expression of PD-L1, which engages the inhibitory receptor programmed-cell death 1 (PD1) on T cells. In fact, the only finding regarding the inhibitory molecules PD1/PD-L1 in chronic infection with *T. cruzi* comes from the murine model in which the blockade of this interaction increased leukocyte infiltration in cardiac tissue and diminished blood parasite load (Fonseca et al., 2018). In accordance, we observed that stimulation with *T. cruzi* induced an increase in PD-L1^+^ B cells in patients with CCD, highlighting that this molecule could protect the heart from an excessive immune response.

**Figure 8:**
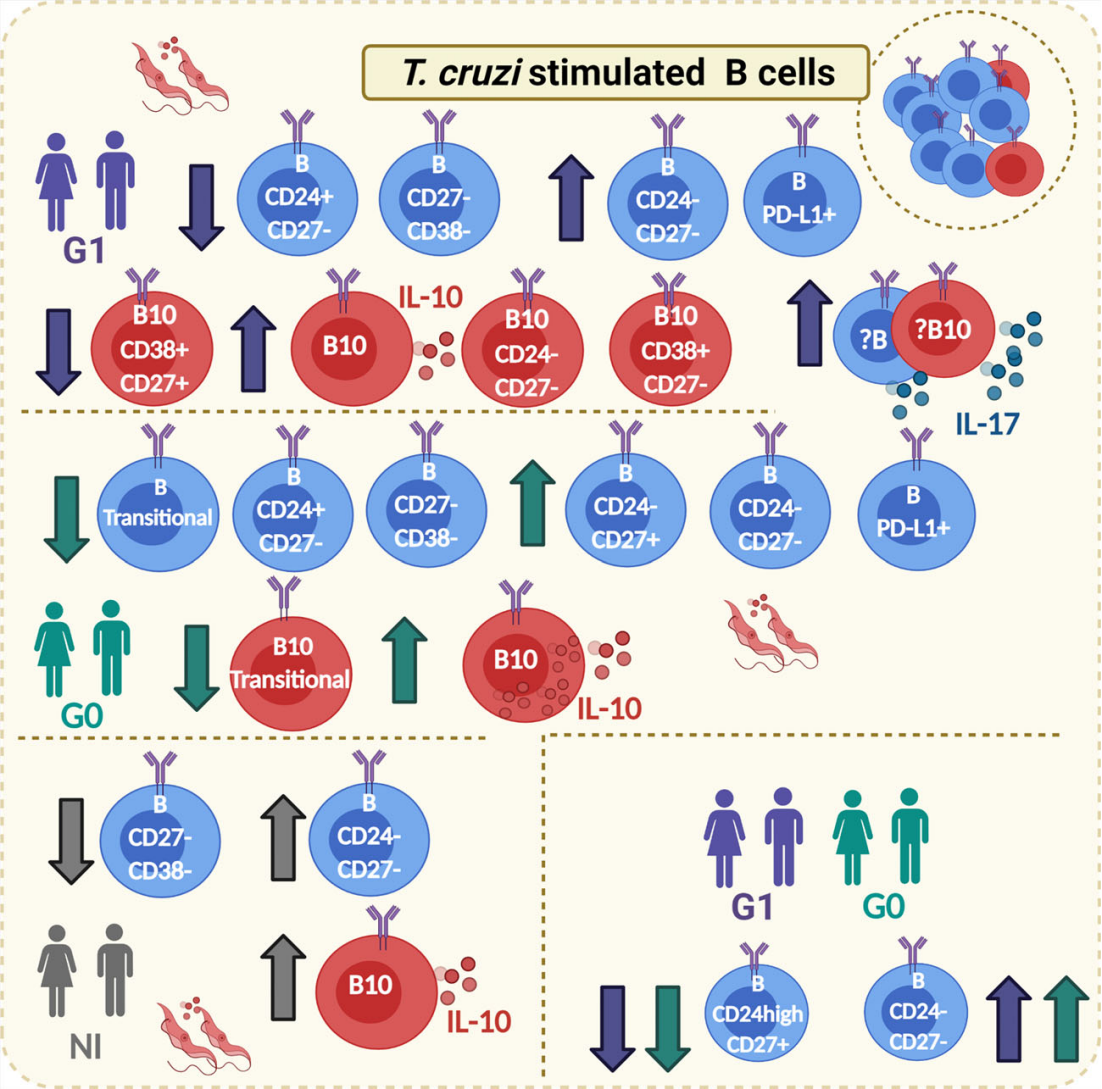
Graphical abstract. Main phenotypic traits identified on B and B10 cells from CCD patients with or without cardiac manifestations and NI donors in response to *T. cruzi* stimulation. Boxes with *T. cruzi* parasite images in red show changes observed on *T. cruzi*–stimulated B cells, whereas the lower box on the right corresponds to changes on subpopulations of B cells that were independent of the stimulation condition. The direction of change is indicated with arrows (increase/decrease). The color of the arrows indicates in which groups of subjects was detected the mentioned alteration (green arrows: G0; purple arrows: G1; gray arrows: NI). Full circles in the cell cytoplasm indicate increased MFI for IL-10, while full circles outside the cell cytoplasm indicate increased secretion of the corresponding cytokine (IL-10 or IL-17). Created with Biorender.com.

The study has some potential limitations. First, the number of subjects included in each group was small. Second, the inability to recruit patients with different cardiac disorders, means stratified as K2 and K3 according to Kuschnir classification, restrained to analyze how the phenotypic traits of B and B10 cells vary with the progression of CCD. Third, the low quantity of B cells isolated per individual limited our ability to measure other anti-inflammatory cytokines such IL-35 and TGF-β, as well as cell surface proteins like TIM-1, and CD73, which would help to unravel unknown aspects of the regulatory mechanisms raised by B cells in patients with chronic Chagas disease. Here, we have detailed the phenotypic characteristics of B and B10 cells based on the expression of CD24, CD27, and CD38 that provides evidence about the potential role of Breg cells in the immune response developed against *T. cruzi*, illustrated in **Figure 8**. Taken together, our findings mainly point out that the frequency of the most representative Breg cells in humans, CD19^+^CD24^high^CD38^high^ and CD19^+^CD24^high^CD27^+^ B cells, are altered in patients with CCD; while both phenotypes dropped in subjects without symptoms, only the latter decreased in patients with cardiac manifestations. Further research is required to uncover the functional effects exerted by these cells to regulate other immune cells within the complex response against *T. cruzi*.

## Data Availability Statement

The raw data supporting the conclusions of this article will be made available by the authors, without undue reservation.

## Ethics Statement

All experimental procedures have been carried out following the standard laboratory biosecurity guidance established by the Instituto de Investigaciones en Ingeniería Genética y Biología Molecular (INGEBI-CONICET).

Subjects were recruited at Instituto Nacional de Parasitología “Dr. Mario Fatala Chabén” under the guidance of Marisa Fernandez, MS, and Yolanda Hernández-Vásquez, MS (Study Protocol N° 3-2018 and 19-2019), and at Hospital General de Agudos “Dr. Ignacio Pirovano,” under the supervision of Raúl Chadi, MS (Study Protocol N° 56-2015). The sampling process followed the tenets of the Declaration of Helsinki and of both institutions’ medical Ethics Committees. Written informed consent was provided by all of the participants.

## Author Contributions

MG designed and performed the experiments, analyzed the data, and wrote the manuscript. MO performed PCR for *T. cruzi* detection. AM-C carried out the parasite culture to produce parasite lysate. MF, YH-V, and RC recruited and classified donors and collected blood samples. KG supervised and designed the research and wrote the manuscript. All authors contributed to the article and approved the submitted version.

## Funding

This research was supported by grants from Consejo Nacional de Investigaciones Científicas y Técnicas (CONICET; PIP Number 112-2015010-0547) and Agencia Nacional de Promoción Científica y Tecnológica, Argentina (ANPCyT; PICT Number 2014-1026). The funders had no role in study design, data collection and interpretation, or the decision to submit the work for publication.

## Conflict of Interest

The authors declare that the research was conducted in the absence of any commercial or financial relationships that could be construed as a potential conflict of interest.

## Publisher’s Note

All claims expressed in this article are solely those of the authors and do not necessarily represent those of their affiliated organizations, or those of the publisher, the editors and the reviewers. Any product that may be evaluated in this article, or claim that may be made by its manufacturer, is not guaranteed or endorsed by the publisher.
