# Supplementary material for: Trypanosoma cruzi Induces Regulatory B Cell Alterations in Patients With Chronic Chagas Disease
Source: Front Cell Infect Microbiol. 2021 Aug 12;11:723549. doi: 10.3389/fcimb.2021.723549 (PMC8387560; doi:10.3389/fcimb.2021.723549)
Supplement: Supplementary file 1 [file DataSheet_1.pdf]

Trypanosoma cruzi induces regulatory B cell alterations in patients with chronic Chagas disease.

Magalí C. Girard, Micaela S. Ossowski, Arturo Muñoz-Calderón, Marisa Fernández, Yolanda Hernández-Vásquez, Raúl Chadi, Karina A. Gómez.

Supplementary Material

1 Supplementary Figures and Tables

1.1 Supplementary Figures

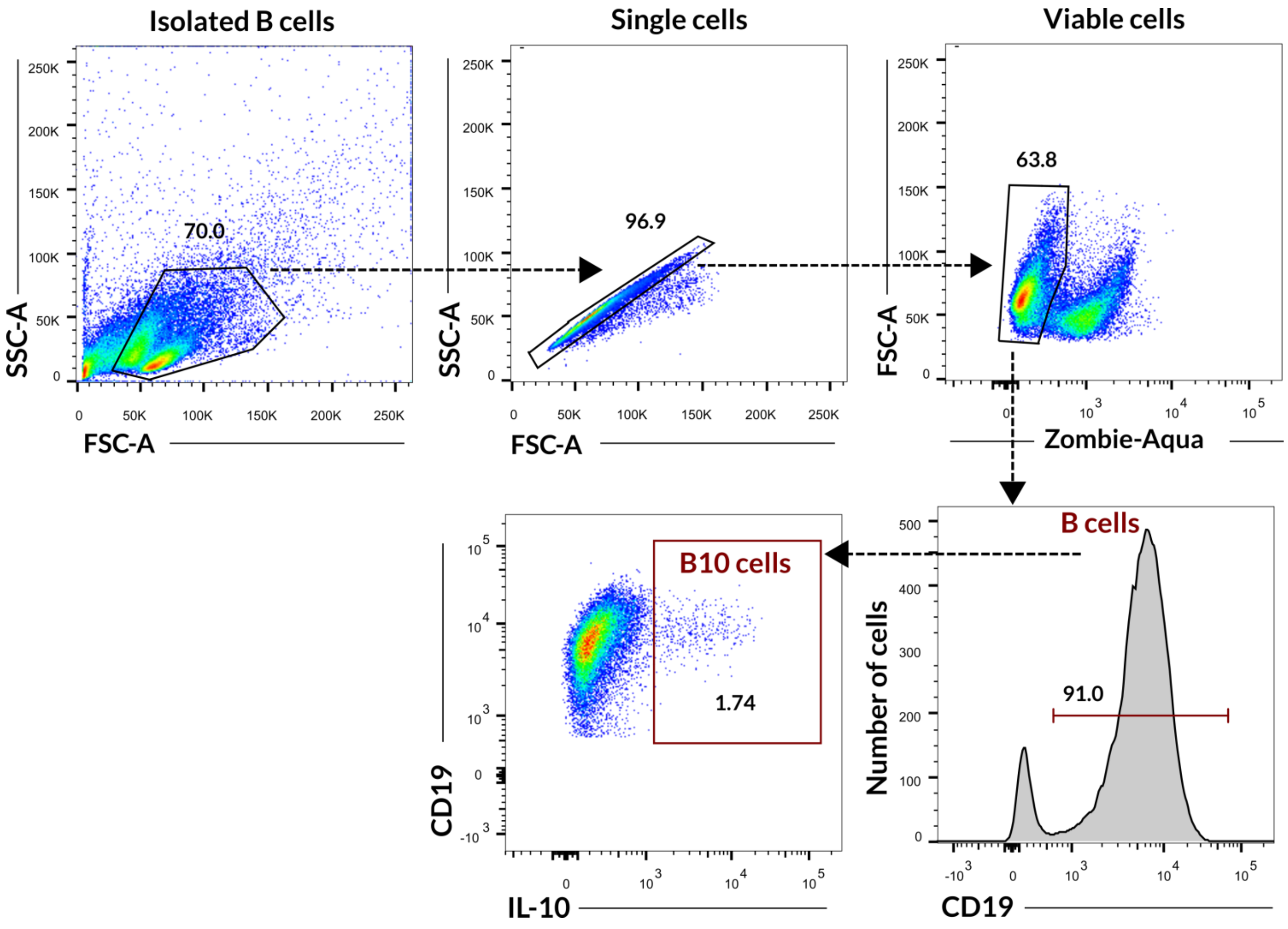

Supplementary Figure 1. Flow cytometry gating strategy for identifying Breg cells from CCD patients and NI donors. The gating strategy employed for each sample is illustrated in a representative NI donor. The sequential scheme shows isolated B cells according to forward scatter/side scatter area (FSC-A/SSC-A) criteria and single cells by gating on the FSC-A/FSC-H

channels. Non-viable cells were excluded by gating on zombie-aqua viability dye channel. Subsequently, B and B10 cells were gated by CD19 and IL-10 expression. Isotype controls were used to determine positive and negative populations for each marker. IL-10^{+} B cells were selected according to subject-matched fluorescence-minus-one (FMO) controls. Further gating analysis of B and B10 cells based on CD24, CD27, CD38 and PD-L1 markers expression is shown in the corresponding Figures. Data analysis was performed using FlowJo software V10.

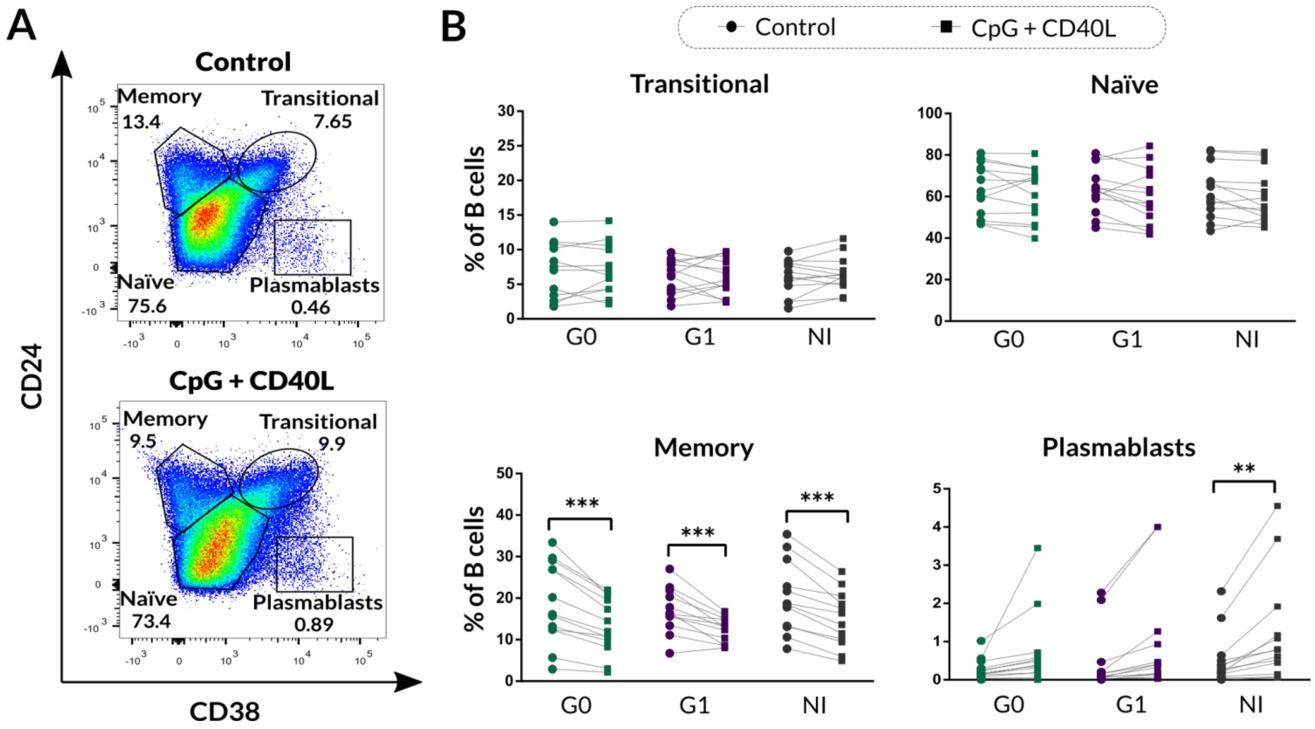

Supplementary Figure 2. Phenotypic distribution based on CD24 and CD38 expression of isolated B cells from patients with CCD and NI donors upon CpG + CD40L stimulation. (**A**) Gating strategy used to identify B cell subsets according to CD24 and CD38 expression. Dot plots from one NI donor are illustrated. (**B**) Frequency of transitional, naïve, memory and plasmablasts subsets in Control or CpG + CD40L stimulated B cells from CCD patients (G0, G1) and NI donors. Each symbol represents data from one individual. Statistically significant differences are indicated with asterisks (**) p<0.01, (***) p<0.001.

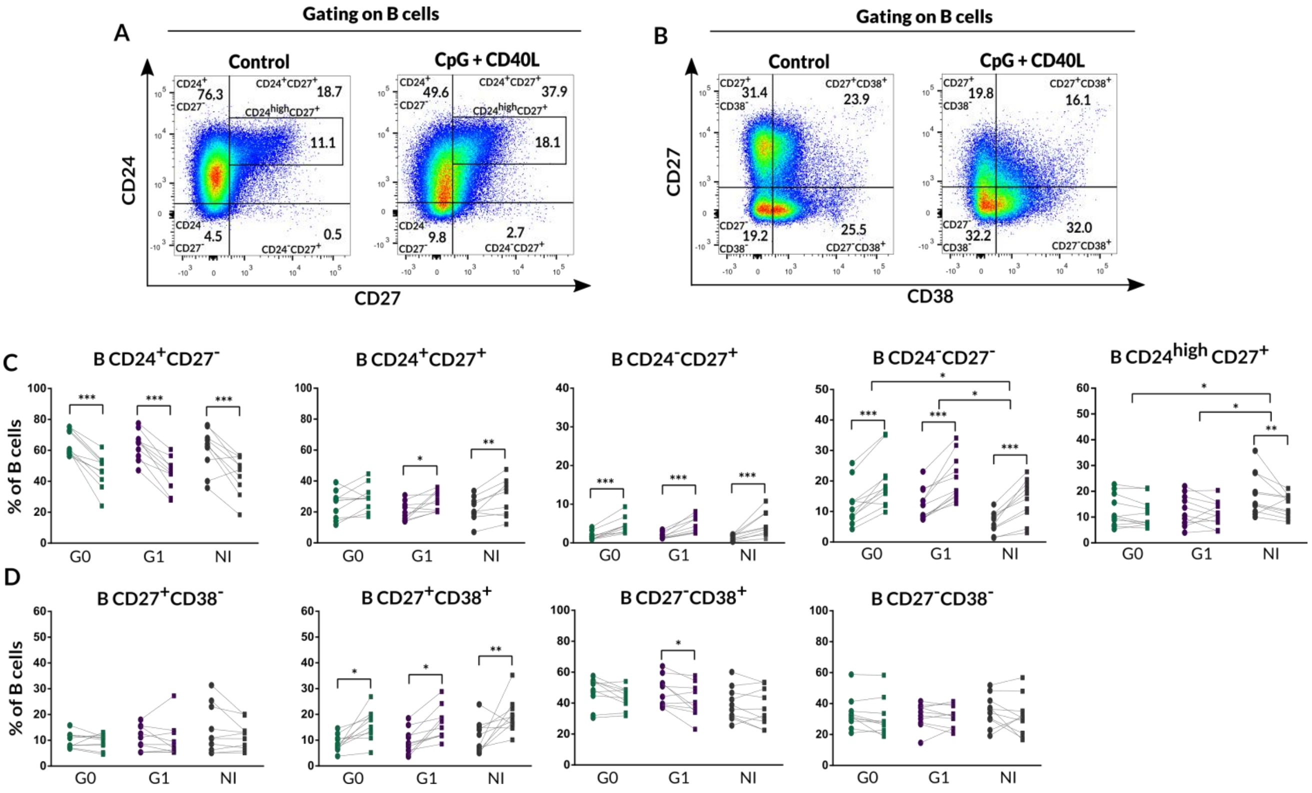

Supplementary Figure 3. Phenotypic distribution based on CD24-CD27 and CD27-CD38 markers expression of isolated B cells from CCD patients and NI donors upon CpG+CD40L stimulation. (**A, B**) Dot plots from one NI donor showing the gating strategy used to identify B-cell subsets defined by CD24-CD27 and CD27-CD38 expression, respectively. (**C, D**) Frequencies of B cell subpopulations in CCD patients (G0, G1) and NI donors in Control or CpG + CD40L stimulated samples according to CD24-CD27 and CD27-CD38 markers expression, respectively. Each symbol represents data from one individual. Statistically significant differences are indicated with asterisks (*) $p<0.05$, (**) $p<0.01$, (***) $p<0.001$.

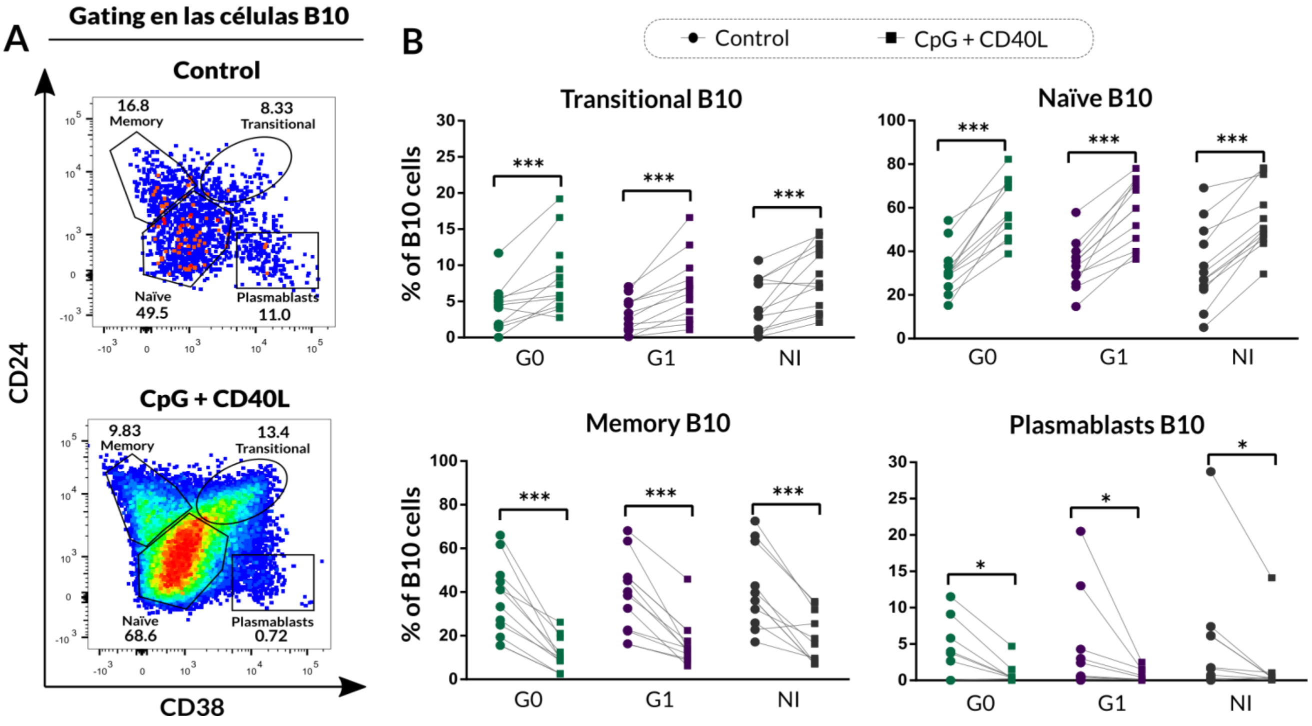

Supplementary Figure 4. Phenotypic distribution of B10 cells based on CD24 and CD38 expression from patients with CCD and NI donors upon CpG + CD40L stimulation. (**A**) Dot plots showing the gating strategy used to identify B10-cell subsets according to CD24 and CD38 expression for one representative NI donor. Large dots were used to facilitate visibility of events. (**B**) Frequency of transitional, naïve, memory and plasmablasts B10 cell subsets in Control or CpG + CD40L stimulated B cells from CCD patients (G0, G1) and NI donors. Each symbol represents data from one individual. Statistically significant differences are indicated with an asterisk (*) $p<0.05$, (***) $p<0.001$.

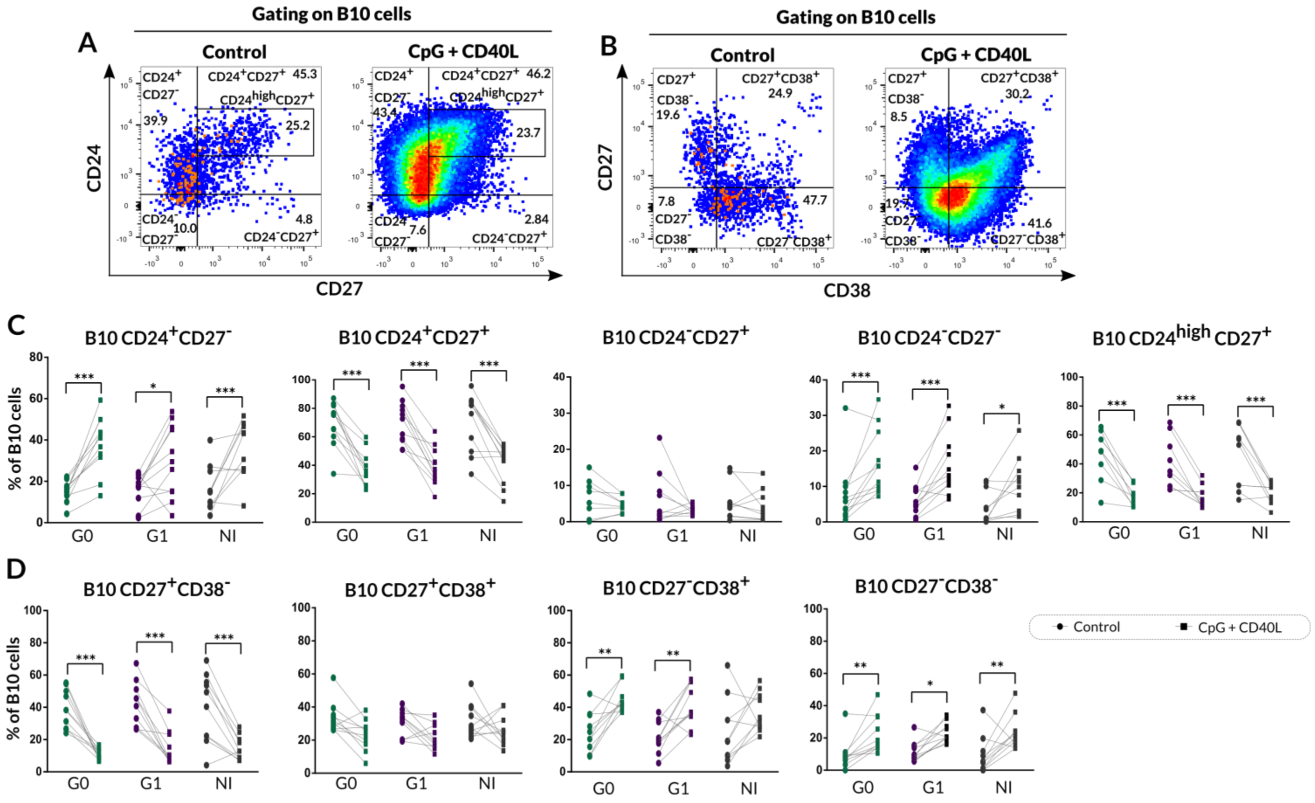

Supplementary Figure 5. Phenotypic distribution of B10 cells in terms of CD24-CD27 and CD27-CD38 markers expression from CCD patients and NI donors upon CpG + CD40L stimulation. (**A, B**) Dot plots from one NI donor showing the gating strategy used to identify B-cell subsets defined by CD24-CD27 and CD27-CD38 expression, respectively. (**C, D**) Box plots show frequencies of B cell subpopulations according to CD24-CD27 and CD27-CD38 markers expression respectively in CCD patients (G0, G1) and NI donors in Control or CpG + CD40L stimulated samples. Each symbol represents data from one individual. Statistically significant differences are indicated with asterisks (*) $p<0.05$, (**) $p<0.01$, (***) $p<0.001$.
